# Clinical and Imaging Prognosis in Patients with Delayed Encephalopathy after Acute Carbon Monoxide Poisoning

**DOI:** 10.1155/2020/1719360

**Published:** 2020-12-07

**Authors:** Jinzhi Liu, Zhihua Si, Jie Liu, Yan Lin, Jing Yuan, Shan Xu, Yan He, Tao Zhang, Aihua Wang

**Affiliations:** ^1^Department of Neurology, Shandong Provincial Qianfoshan Hospital, The First Affiliated Hospital of Shandong First Medical University, Jinan, China; ^2^Department of Neurology, Shandong Provincial Qianfoshan Hospital, Shandong University, Jinan, China

## Abstract

**Objective:**

To study the clinical manifestations, magnetic resonance imaging (MRI) findings, and prognosis of delayed encephalopathy after carbon monoxide poisoning (DEACMP).

**Methods:**

The medical records of 20 patients with DEACMP were retrospectively reviewed. All the patients received hyperbaric oxygen treatment and other treatments as necessary.

**Results:**

The patients had diverse clinical manifestations, including memory deficits, personality changes, cognitive or executive function deficits, mood disorders, Parkinsonism, dystonia or other motor impairments, and akinetic mutism. MRI revealed lesions in the bilateral cerebral white matter and/or basal ganglia. Except for the pathologically confirmed DEACMP, epileptic seizure, hemiplegia, and vegetative state, the remaining symptoms had been improved, especially the cognitive impairment, which had been decreased from 95% to 25% and psychiatric symptoms also decreased from 95% to 55% at the 6-month follow-up.

**Conclusions:**

The prognosis of patients with DEACMP was poor, and they had a relatively severe disability. The early use of hyperbaric oxygen is of great significance to improve clinical efficacy and get a better prognosis.

## 1. Introduction

Carbon monoxide (CO) is a type of colorless, tasteless, odorless, and nonirritating gas with toxicity, resulting from the incomplete combustion of carbon-based fuels [1]. CO poisoning is a leading contributor to deaths from poisoning worldwide, including the United States, China, Japan, and Europe, Turkey [2–6]. Global epidemiologic data reveal that the accumulative morbidity and mortality of CO poisoning are approximately 137 cases and 4.6 deaths per million throughout the world [7]. Furthermore, it has been reported that the incidence of CO poisoning is higher in developing countries like China than in developed countries such as the United States due to the wide application of nonclean energy [8]. Of note, CO poisoning is the most common poisoning in northern China, as most people rely on coal burning for heat in the cold season [9]. The mechanism underlying CO poisoning is that the affinity of CO and hemoglobin (Hb) is 200-300 times stronger than that of oxygen and hemoglobin, resulting in systemic tissue hypoxia [10]. Studies have shown that hypoxia-caused CO in the brain produced too many oxygen free radicals, which easily damaged the nerve cells, leading to CO poisoning symptoms.

Delayed encephalopathy after carbon monoxide poisoning (DEACMP) is a group of neuropsychological disorders that can occur days or weeks after the cessation of symptoms of acute CO poisoning [11–13]. Previous studies have indicated that 0.2%-40% of CO poisoning patients develop DEACMP. Serious DEACMP is usually fatal and as many as 50% of DEACMP individuals develop cognitive, neurological, or neurobehavioral sequelae [14]. After the false recovery periods, patients with acute CO poisoning symptoms reappear including neurological symptoms, cognitive disorders, mental disorders, and pyramidal or extrapyramidal symptoms. Common neurological manifestations of DEACMP include disorders such as computational power and memory loss, linguistic disorders, dystonia, orientation disorders, dementia, Parkinson's syndrome, and kinetic mutations [15]. The changes of CT and MRI were consistent with the pathological basis. The abnormality rate of CT in the brain was about 87.5%, which was manifested as the diffuse abnormal signal changes of the bilateral globus palidus, forehead, dorsal occipital brain hemisphere white matter and bilateral basal nuclear region low-density changes, lateral ventricle expansion, sulcus widening, and brain tissue atrophy. The main performance of MRI was shown as follows: (1) subcortical white matter abnormalities: mainly under the cortical point, patchy or fusion-like T1WI low signal and T2WI high signal; (2) deep white matter lesions: the periventricular white matter and semioval center, the more extensive T2WI low signal and T2WI high signal; and (3) basal ganglia lesions: bilateral white-white symmetrical T1WI low signal and T2WI high signal [12, 16–18].

To better understand the clinical course of DEACMP and lessen the associated disability, this study investigated the clinical characteristics, MRI findings, and prognosis of 20 patients with DEACMP.

## 2. Materials and Methods

### 2.1. Patients

This retrospective study was approved by the Ethics Committee of Shandong Provincial Qianfoshan Hospital, China (NO. EAFQ2012022). Written informed consent was obtained from all the patients or their relatives between January 2012 and January 2013. From January 2010 to January 2013, 100 patients with toxicity were admitted to the emergency department of our hospital. Among these, 20 patients, 12 males and 8 females, with the mean age of 61 years fulfilled the following inclusion criteria: (1) a clear history of CO poisoning, resulting in a coma, in the previous 1-2 months; (2) normal or nearly normal performance in the interim period (i.e., between recovering from the immediate coma caused by CO poisoning and the occurrence of clinical manifestations of DEACMP); (3) clinical features of acute dementia or full-brain damage, including advanced neurological deficits, psychiatric symptoms, pyramidal system disorders, and extrapyramidal manifestations, coupled with peripheral and cranial nerve damage; and (4) no other intracranial lesions.

MRI was performed on the first day of hospitalization using a 3-T magnet (GE Signa HDx 3 T) [12]. T1-weighted images (repetition time, 1770 ms; echo time, 9.0 ms), T2-weighted images (repetition time, 4400 ms; echo time, 100 ms), and T2-weighted FLAIR images (repetition time, 7000 ms; echo time, 86 ms) were obtained in the coronal, sagittal, and axial planes. Only patients with prominent leukoencephalopathy but no subclinical leukoencephalopathy were recruited.

### 2.2. Classification of cognitive impairment

Cognitive performance and functional state were assessed before and after treatment by the neuropsychologists. The classification is as follows:

1. Mild: mild injury of brain tissue, decline of computing power, memory and understanding ability, abnormal language and action, and poor self-care ability
2. Moderate: in addition to mild performances, heavier injuries of the central nervous system or other vital organs and tissues, and incoherent speech, sudden abuse, shouting, severe depression, hemiplegia, urinary incontinence, and other symptoms
3. Severe: severe damage of the central nervous system, the patient presented with a coma or even persistent vegetative state

### 2.3. Treatment

All the patients underwent at least one session of hyperbaric oxygen therapy (2.5-3.0 absolute atmospheres (atm) for ≥90 min). The pressure was increased from 1 atm to 2.8 atm over the first 18 min, maintained at 2.8 atm for 50 min, and then decreased to 1 atm over 22 min. This procedure was carried out five times a week. Supportive therapy was provided when necessary, including the administration of edaravone, a free radical scavenger, and selegiline, a monoamine oxidase inhibitor. The period of hospitalization ranged from 20 to 30 days, depending on the clinical condition of the patient.

## 3. Results

### 3.1. Clinical Characteristics and Follow-Up Prognosis

The main clinical characteristics were shown in Table 1. Nineteen patients (95%) exhibited a cognitive impairment before clinical treatments. Based on the clinical classifications, the condition of DEACMP patients was assessed as slight (3 patients), moderate (9 patients), and severe (7 patients). Three patients developed generalized tonic-clonic seizures, and one patient developed simple partial seizure (Table 1).

After 6 months of follow-up, we found that except for the pathologically confirmed DEACMP, epileptic seizure, hemiplegia, and vegetative state, the remaining symptoms had been improved in all patients (Table 1). Among them, the cognitive impairment decreased from 95% to 65% (*n* = 2 slight, *n* = 7 moderate, and *n* = 4 severe) at 1-month follow-up, to 40% (*n* = 2 slight, *n* = 4 moderate, and *n* = 2 severe) at 3-month follow-up, and to 25% (*n* = 3 slight, *n* = 2 moderate, and *n* = 0 severe) at 6-month follow-up. In addition, psychiatric symptoms also decreased from 95% to 90% at 1-month follow-up, to 80% at 3-month follow-up, and to 55% at 6-month follow-up.

### 3.2. Imaging Findings

**Figure 1:**
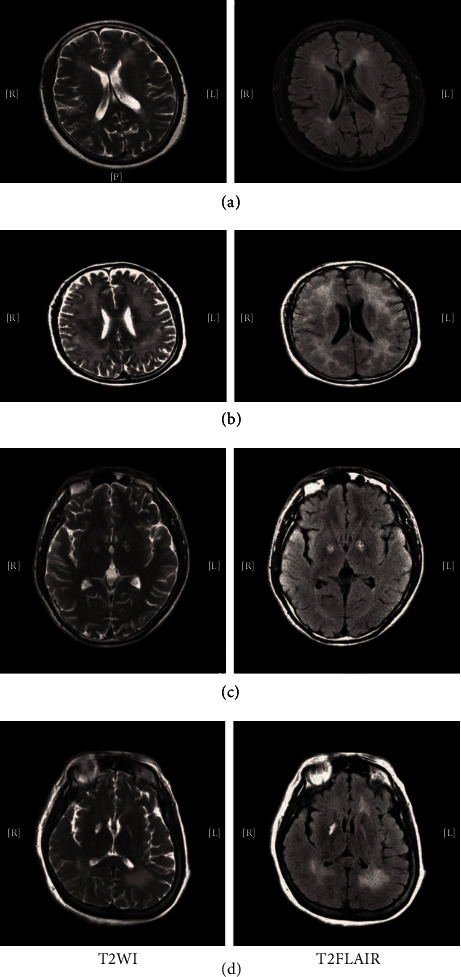
magnetic resonance imaging (MRI) scan showed high signal intensity in the bilateral cerebral white matter (a, b) and the globus pallidus(c, d).

The T2-weighted images and T2-weighted FLAIR images indicated high signal intensity in the bilateral cerebral white matter and bilateral or unilateral globus pallidus and periventricular white matter (Figure 1). There were 7 patients (35%) with high signal intensity in the bilateral cerebral white matter, 2 cases (10%) displaying basal ganglion lesions, and 11 patients (55%) with basal ganglion lesions accompanied by the periventricular white matter and semielliptical central lesions.

## 4. Discussion

Two courses generally following exposure to CO are acute poisoning that occurs immediately after exposure to CO or acute poisoning complicated by delayed encephalopathy following a symptom-free interval of a few weeks or more [14]. Our data revealed that the occurrence of DEACMP accounted for about 20% in patients who underwent CO poisoning caused by unintentional accidents, which was consistent with previous studies [19, 20]. In this study, we reported the clinical characteristics of 20 patients with DEACMP and their associated neuroradiological changes. Before clinical treatment, 95% of the patients suffered from cognitive impairment and 100% had functional impairment. Except for the pathologically confirmed DEACMP, epileptic seizure, hemiplegia, and vegetative state, the remaining symptoms had been improved, especially the cognitive impairment had been decreased from 95% to 25% and psychiatric symptoms also reduced from 95% to 55% at the 6-month follow-up.

Although the pathogenesis of DEACMP is not clear, it is generally believed that (1) vascular wall degeneration, vascular motor nerve palsy, vasodilation, congestion, vascular rupture, and occlusive arterial endocarditis can result in brain cell necrosis, softening, and degeneration; (2) oligodendrocytes are vulnerable to hypoxia, thereby triggering extensive demyelination, which is important for the disease progression; (3) immune dysfunction, abnormalities of dopamine, serotonin, acetylcholine, and other neurotransmitters and free radical-induced lipid peroxidation are involved in the occurrence and progress of the disease [21–24].

Hyperbaric oxygen treatment, as the most effective clinical therapy, of acute toxicity of CO poisoning, has a long history, which can significantly improve the prognosis of patients and reduce the incidence of DEACMP. Hyperbaric oxygen therapy can increase the physical dissolved oxygen content for the body to provide adequate oxygen metabolism without relying on HbO_2_ dissociation. With the increase of the oxygen supply, the vascular wall permeability can come back to normal, which may reduce oozing, cerebral edema, and intracranial pressure; promote the establishment of collateral circulation, and reduce anaerobic glycolysis, blood viscosity, and platelet aggregation. The incidence of DEACMP is usually around 12%-13% in patients who have been poisoned for more than 6 hours or who have not received hyperbaric oxygen therapy. In patients receiving hyperbaric oxygen therapy within 6 hours following poisoning, the incidence rate is approximately 1% [14, 25, 26]. However, little research has been reported about the use of hyperbaric oxygen therapy for DEACMP. Several studies suggest that the duration of coma, COHb saturation, and hyperbaric oxygen may be the risk factors. Besides, it has been demonstrated that there is a certain correlation between the clinical symptoms of DEACMP and the imaging characteristics [1, 11, 17]. When the cortical white matter shows abnormal signals, the clinical manifestations may include cognitive dysfunction and language disorders, accompanied by anxiety, depression, and other psychiatric symptoms. And the imaging of the basal ganglia lesions or basal ganglia with periventricular white matter and semioval center lesions may be not only associated with the cognitive dysfunction but also correlated with the accompanied nonvascular disease, dystonia, and other Parkinson's syndrome. DEACMP occurs in the white matter of the brain, causing demyelination [12, 17, 24]. We also deem that the cerebral hypoxia can cause the secondary microvascular injury, cell swelling, and degeneration; deep or intravenous congestion of the microvascular wall; and vascular occlusion, ultimately leading to brain cell necrosis, white matter loose, etc. Our study suggests that the treatment of DEACMP with hyperbaric oxygen therapy combined with hormones, circulations, and blood circulation therapy as soon as possible may improve the symptoms of the poisoning based on the diagnosis. It is worth noting that, in our study, there are more patients with basal ganglion lesions accompanied by the periventricular white matter and semielliptical central lesions when compared to those with simple basal ganglia lesions, which is related to the severity of the disease in the cases received by our hospital. Meanwhile, patients with simple basal ganglia lesions mainly present dystonia and Parkinson-like symptoms, which is associated with the low rate of consultation and hospitalization.

It is widely acknowledged that the efficacy of hyperbaric oxygen may be related to the age, the disease classification, and the treatment [20, 21, 27]. Even so, we recommend that the patients should be actively applied with hyperbaric oxygen treatment regardless of the poisoning phase and they should not give up the hyperbaric oxygen therapy until the real brain death is diagnosed. In fact, some patients with the cognitive, neurological, or neurobehavioral sequelae may return to normal function after DEACMP for a long time. A prospective study is needed to analyze the long-term prognosis of the DEACMP patients.

## 5. Conclusions

DEACMP patients have severe neurological damage and poor prognosis. From this study, we consider that the early use of hyperbaric oxygen is of great significance to improve clinical efficacy and get a better prognosis according to the analysis of high-risk factors combined with clinical symptoms and related checks. It is vital for individuals to take precautions to minimize potential exposure to carbon monoxide in the home.

## Figures and Tables

**Table 1:** Clinical characteristics and follow-up prognosis.

Symptoms	Cases	Follow-up 1 m	Follow-up 3 m	Follow-up 6 m
Cognitive impairment	19 (95%)	13 (65%)	8 (40%)	5 (25%)
Urinary incontinence	14 (70%)	14 (70%)	12 (60%)	11 (55%)
Psychiatric symptoms	19 (95%)	18 (90%)	16 (80%)	11 (55%)
Pathologically confirmed DEACMP	6 (30%)	6 (30%)	6 (30%)	6 (30%)
Extrapyramidal symptoms	18 (90%)	18 (90%)	17 (85%)	15 (75%)
Mutism	18 (90%)	18 (90%)	17 (85%)	16 (80%)
Epileptic seizure	4 (20%)	4 (20%)	4 (20%)	4 (20%)
Pseudobulbar palsy	16 (80%)	16 (80%)	15 (75%)	12 (60%)
Hemiplegia	3 (15%)	3 (15%)	3 (15%)	3 (15%)
Vegetative state	11 (55%)	11 (55%)	11 (55%)	11 (55%)

The data were shown as *n* (percentage). DEACMP: delayed encephalopathy after carbon monoxide poisoning.

## Data Availability

The analyzed data sets generated during the study are available from the corresponding author on reasonable request.
